# Alzheimer’s disease causation by copper toxicity and treatment with zinc

**DOI:** 10.3389/fnagi.2014.00092

**Published:** 2014-05-16

**Authors:** George J. Brewer

**Affiliations:** Department of Human Genetics, University of Michigan Health SystemAnn Arbor, MI, USA

**Keywords:** Alzheimer’s disease, inorganic copper, serum free copper, zinc deficiency, cognition

## Abstract

Evidence will be presented that the Alzheimer’s disease (AD) epidemic is new, the disease being very rare in the 1900s. The incidence is increasing rapidly, but only in developed countries. We postulate that the new emerging environmental factor partially causal of the AD epidemic is ingestion of inorganic copper from drinking water and taking supplement pills, along with a high fat diet. Inorganic copper can be partially directly absorbed and elevate the serum free copper pool. The Squitti group has shown that serum free copper is elevated in AD, correlates with cognition, and predicts cognition loss. Thus, our inorganic copper hypothesis fits well with the Squitti group data. We have also shown that AD patients are zinc deficient compared to age-matched controls. Because zinc is a neuronal protective factor, we postulate that zinc deficiency may also be partially causative of AD. We carried out a small 6 month double blind study of a new zinc formulation and found that in patients age 70 and over, it protected against cognition loss. Zinc therapy also significantly reduced serum free copper in AD patients, so efficacy may come from restoring normal zinc levels, or from lowering serum free copper, or from both.

## Introduction

Cancer, heart disease, and stroke have increasingly become major killers in our Western societies. We have looked for environmental factors behind the increased mortality from these diseases, and we have found them. They include cigarette smoking, air pollution, diets high in fats and sugars and lacking in fruits and vegetables, and lack of exercise, to name but a few. Considerable effort has gone into identifying and mitigating the environmental factors causing so much mortality from these diseases.

In stark contrast, a new disease epidemic has crept into our midst, clearly also strongly caused by environmental factors, but almost no effort has been made to find the environmental culprits and mitigate them. Perhaps this is because this new disease does not quickly kill, rather it only robs a large segment of our elderly of their ability to function effectively. This new disease epidemic is Alzheimer’s disease (AD), and its close cousin, mild cognitive impairment (MCI).

Why do we say it’s a new epidemic? First, it is an epidemic, with 10% of those aged 60, 20% of those aged 70% and 30% of those aged 80 in the U.S. affected with the disease (Alzheimer’s Association, 2010).There are over 5 million Americans with AD, with an equal number with MCI, 80% of whom develop AD at the rate of 15% per year. Second, it is new, because it didn’t exist or was very rare prior to 1900, and developed a rapidly increasing prevalence after about 1950.

The evidence that AD didn’t exist, or was rare, prior to 1900 is quite good. In their book, Dying for a Hamburger, Waldman and Lamb (Waldman and Lamb, 2005) examined this question. They point out that Osler, a clinician who edited a textbook pulling together all known diseases in the late 1800s, did not mention an AD like disease, although one entire volume was devoted to diseases of the brain (Osler, 1910). Gowers, who wrote a textbook of neurology during this period, also did not describe an AD like disease (Gowers, 1888). Most important, Boyd, who wrote a textbook of pathology during the late 1800s, updated in the early 1900s, did not describe amyloid plaques neurofibrillary tangles, hallmarks of AD brain pathology, in brains at autopsy (Boyd, 1938).

Some say that since AD is a disease of aging, there just were not enough old people around in the period of the 1800s, so the disease was not observed, or at least not noticed. It is not true that elderly people weren’t around back then. Waldman and Lamb showed that in 1911, half the French population were living to age 60, the age of onset of AD (Waldman and Lamb, 2005). I checked the US census for 1900, and there were 3.2 million people over age 60, at today’s rate providing 36.3 thousand AD cases, more than enough to have been frequently seen in the clinics of Osler and Gower, and to have been frequently encountered at autopsy by Boyd.

Others say that the disease does not represent a “new” epidemic because now our ability to recognize it as a disease is much better, in other words our “diagnostic ability” is now greatly improved. While this could conceivably explain the failure of clinicians, such as Osler and Gowers, to recognize the disease, although this seems unlikely given their thoroughness, it can’t explain why pathologists such as Boyd didn’t observe amyloid plaques and neurofibrillary tangles in brains at autopsy.

So one fact is we have a new epidemic. The second fact is that the epidemic is primarily occurring in developed countries (except for Japan), and not in undeveloped countries (Ferri et al., 2005).

These two facts make it clear that something or some things, newly present in the last 100 years in the environment of developed, but not undeveloped, countries is causing this epidemic of AD. Waldman and Lamb come to this same conclusion, and because they believed the new factor was beef eating and AD was a prion disease acquired from beef, they named their book, Dying for a Hamburger (Waldman and Lamb, 2005). Certainly beef eating fits the criteria of being associated with development over the last 100 years, but we find no evidence that AD is a prion disease. Nevertheless, we believe Waldman and Lamb were on the right track, because we believe a high fat diet is one causal factor for the AD epidemic, and beef eating is associated with a high fat diet. Grant has shown that AD prevalence is positively correlated with dietary fat intake across many countries (Grant, 1997). However, we believe an additional factor plays a major causal role for the AD epidemic. For our view on this additional factor, read on.

## Our hypothesis that intake of inorganic copper is a major causal factor in the ad epidemic

The major stimulus to our awareness that ingestion of inorganic copper could be a risk factor for AD resulted from the studies of Sparks and Schreurs, reported in 2003 (Sparks and Schreurs, 2003). In studies of a cholesterol fed rabbit model of AD, they found that addition of as little as 0.12 ppm copper to the distilled drinking water of the rabbits greatly enhanced both AD type brain pathology, and cognition loss by the animals. By reference, the Environmental Protection Agency in the U.S. allows up to 1.3 ppm copper in human drinking water, over 10 times that causing toxicity in the rabbit AD model. Allowances for copper in drinking water are similar, or higher, around the world. The work of Sparks and Schreurs has been confirmed by them in other AD models, including the mouse model (Sparks et al., 2006), and have been confirmed by another group (Singh et al., 2013).

We emphasize that we are talking about ingestion of inorganic copper as being toxic, not organic copper. Organic copper is copper in food, safely bound to proteins. Organic copper is absorbed from the intestine into the blood, and processed by the liver, and put into safe channels. In contrast copper in drinking water (or in pills containing copper) is a simple inorganic salt of copper, not bound to anything. We have evidence that at least a portion of inorganic copper is absorbed into the blood, bypasses the liver, and contributes immediately to the “free copper” pool in the blood (Hill et al., 1986). A small increase in copper in food, equivalent to 0.12 ppm, would be trivial and completely non-toxic, whereas it is exquisitely toxic in drinking water, as shown in the rabbit model.

The size of the “free copper” pool in the blood is very important in AD, as shown by Squitti et al. (2005). Depending on how it is measured, about 65–85% of the copper in blood is covalently bound in ceruloplasmin (Cp), a copper containing protein secreted into the blood by the liver, and is safe copper. The remainder is loosely bound to albumin and some other molecules, and is called “free copper”. The Squitti group has shown that blood free copper levels in AD are significantly elevated compared to age matched controls (Squitti et al., 2005), that free copper levels are inversely correlated with cognition measure in AD (the higher the free copper, the worse the cognition measures) (Squitti et al., 2006), and are positively correlated with loss of cognition over time (the higher the free copper, the greater the loss of cognition) (Squitti et al., 2009). Thus, it is very rational that addition to the free copper pool by ingestion of inorganic copper, because some of it immediately increases the free copper pool, could be a risk factor for AD causation.

The level of total plasma copper in AD patients versus age matched controls should not be confused with the levels of blood free copper we first discussed. Some authors have found total serum copper elevated in AD, while others have not. But the relevant finding is that blood free copper is elevated in AD.

So ingestion of inorganic copper in drinking water could be a risk factor in AD, but are humans ingesting inorganic copper in their drinking water? The answer is yes, it is being leached from their copper plumbing! Looking back at the time course of the AD epidemic it is remarkable how closely it parallels the explosive use of copper plumbing in developed countries. Copper plumbing began to be used in the early 20th century, but was curtailed by World War I and then II. After 1950 copper plumbing took off, and now 90% of U.S. homes have copper plumbing. Similarly the AD epidemic took off in the latter half of the 20th century, but only in developed countries. Copper plumbing is not used very much in undeveloped countries, because of its expense. Japan is an interesting exception that supports the copper in drinking water causation hypothesis. It is a developed country, but with a lower rate of AD (Ueda et al., 1992), and has shunned copper plumbing for fear of toxicity. Yet, when Japanese migrate to Hawaii, where copper plumbing is used, they developed the higher rate of AD seen in other developed countries (White et al., 1996).

We stated above that leaching of copper from copper plumbing into drinking water is a risk factor of AD, but what is our evidence that copper is actually leached into drinking water in significant amounts? Our evidence is that we have measured it. We wanted to make sure our patients with Wilson’s disease, a disease of copper toxicity, weren’t ingesting excessive copper in their drinking water. These patients came from all over N. America, attracted to our clinic because of new Wilson’s disease treatments we were developing. In a sample of drinking water from 280 homes, we found about a third were higher than the 0.1 ppm causing toxicity in the rabbit AD model, about one third were 0.01 ppm or lower, a level we view as safe, and about one third were between 0.01 and 0.1 ppm, an area of unknown safety (Brewer, 2011). So people are ingesting plenty of inorganic copper from their drinking water, which according to the rabbit AD model could account for the high and increasing prevalence of AD.

There is another source of inorganic copper leading to high ingestion in the developed world, and that is use of supplement pills containing copper. As with copper in drinking water, the copper in pills is a simple salt, such as copper sulfate, and is thus inorganic. Morris et al. (2006) did a study in Chicago of nutrient intake, and cognition, over a period of years. They found that those in the highest quintile of copper intake, if they also ate a high fat diet, lost cognition at six times the rate of other groups. People were in the highest quintile of copper intake because they took supplement pills containing copper.

One can also build a case for the other risk factors for AD tying into the copper hypothesis. We believe another major risk factor for AD is a high fat diet (Grant, 1997). Copper oxidizes certain fat molecules into derivatives that are toxic to neurons. Elevated homocysteine levels are a risk factor for AD (as they are for atherosclerosis) (Seshadri et al., 2002). Homocysteine interacts with copper to oxidize cholesterol to an intermediate damaging to neurons. The apoliprotein E4 (apoE4) allele is a risk factor for AD, while apoE2 is protective and apoE3 neutral (Miyata and Smith, 1996). Apoliprotein may help remove copper from the brain, and apoE4 has no copper binding cysteines, apoE3 one, and apoE2 two. Certain hemochromatosis (Moalem et al., 2000) and transferrin (Zambenedetti et al., 2003) alleles increase risk for AD. These genes affect iron levels, and iron, like copper, is a transition element that produces oxidant radicals. Oxidant damage is a predominant type of damage in the AD brain. Recently it has been shown that certain alleles of the ATP7B gene, also known as the Wilson’s disease gene, increase risk for AD (Bucossi et al., 2011, 2012). This is further direct support of our hypothesis, because this gene controls free copper levels.

Recently there has been some direct studies that strongly support a critical role for copper toxicity in the AD brain. It has been shown that “labile copper” is elevated in the AD brain, and that this elevation is associated with oxidant pathology in the AD brain (James et al., 2012).

In summary, we believe our hypothesis that ingestion of inorganic copper is a major risk factor for AD is well supported. Recounting the network of supporting data around this hypothesis that constitutes good support, we have the observation that trace amounts of inorganic copper in drinking water greatly enhances AD pathology and cognition loss in AD animal models, we have the concordance of the AD epidemic and use of copper plumbing, the data showing toxic levels of copper in drinking water all over N. America, the data of Morris et al. (2006) showing cognition loss in those using copper supplements, the data of Squitti et al. (2005) showing a high serum free copper in AD patients, the data showing that gene mutations potentially influencing copper levels increase AD risk, and the direct data showing that “labile copper” is elevated in the AD brain.

Some authors have concluded the opposite, that copper deficiency is a causal factor in AD. An example of this is Klevay (2008), who hypothesize just that, that AD is copper deficiency. However, this hypothesis is not well thought out, since AD patients display none of the manifestations of copper deficiency, which are a very low serum copper, anemia and bone marrow depression, and myelopolyneuropathy neurologic syndrome. Another example is Kessler et al. (2008) who gave copper to AD patients, and claimed to see no worsening. They saw no improvement either, which disproved their hypothesis that patients were suffering from copper deficiency. We believe that ingestion of a high fat diet is another important risk factor for AD, and that these two, ingestion of inorganic copper plus a higher fat diet, set the stage on which the other risk factors act.

It is sometimes said that a hypothesis is only useful if it can be tested. It is a little hard to test this one definitively. The AD animal model studies have probably gone as far as one can go with animal studies. It is unethical to give humans potentially toxic inorganic copper. Epidemiologic studies could be designed, but these end up showing associations, which don’t prove causation. Inorganic copper ingestion could be prevented in a large sample of elderly (people age 60 or over), and AD outcomes compared with an equally large sample of controls who continued ingestion of inorganic copper at their normal rates, but this would take years to complete, and be quite expensive. In the meantime, those who believe this hypothesis is likely to be true, would be well-advised to limit ingestion of inorganic copper.

## Our hypotheis that zinc deficiency is a risk factor for ad and cognition can be stablized by zinc supplementation

As people grow older, they become relatively zinc deficient compared to younger people, as measured by serum zinc levels. To evaluate the zinc status of AD patients we did a study on AD patients and age-matched controls with Earl Zimmerman and his group at Albany, NY. Because elderly people take many mineral supplements, to make sure supplements weren’t affecting the results, we stopped all supplements use one month prior to the study. In 29 age-matched controls, mean serum zinc was 82.7 μg/dl, as expected well below that of younger people, which runs around 100 μg/dl. But the mean serum zinc of 29 AD patients was 76.2 μg/dl, significantly less than the mean of age-matched controls (Brewer et al., 2010). Baum et al. (2010) have also found zinc deficiency in serum of AD patients.

The fact that zinc levels are elevated in the AD brain led to early speculation that zinc excess and zinc toxicity was a factor in AD. However, now it is realized that amyloid plaques bind zinc avidly, and this causes an increase brain zinc in AD. The uptake of zinc by plaques is another cause of diminished zinc availability to neurons.

Zinc has many important protective roles in neurons. For example, it helps quench glutamate-stimulated neuronal firing, preventing damage from excessive firing (Takeda, 2010). It also inhibits calcineurin, which if too active, has downstream adverse effects (Crouch et al., 2011). Serum zinc, when it is low, is a reliable indicator of systemic zinc deficiency. But the brain in AD may suffer even more from zinc deficiency than indicated by the serum levels. That is because the amyloid plaques in the AD brain are avid binders of zinc, and may make zinc even less available for neurons.

Additional insight and credibility for the hypothesis that the AD neuron is zinc deficient comes from the studies of the zinc transporter-3 (ZnT3). This transporter is the zinc pump that loads neuronal vesicles with zinc, the vesicles that are discharged into the synapse with neuronal firing. ZnT3 knockout mice exhibit deficits in memory and learning at 6 months of age, and are said to be “a phenocopy of the synaptic and memory deficits of AD” (Adlard et al., 2010). These same authors also found that ZnT3 levels decreased with aging in the brains of mice and humans, and decreased significantly more with aging in the brains of AD patients compared to age-matched controls.

For all these reasons, we hypothesize that zinc deficiency is a risk factor in AD, that is, a factor which can precipitate, or at least assist, cognitive decline. Given this hypothesis, it seemed reasonable to evaluate the possible beneficial effect of zinc supplementation.

In reviewing the literature, it turned out zinc supplementation, both oral and parenteral, was tried in open label studies in 1991 by Constantinidis (1992). He reported excellent efficacy, although the trials were uncontrolled. Corona et al. (2010) have also reported beneficial effects of zinc supplementation in a rodent AD model.

In our trial, we used a new zinc formulation developed by myself and a company, Adeona Pharmaceuticals. I had previously developed zinc acetate capsules, called Galzin, and had them FDA approved for treating Wilson’s disease. The two problems with Galzin were, first, it had to be taken away from food and second, it had to be taken multiple times a day if one wanted to achieve around the clock plasma zinc elevations. The new zinc formulation employed a zinc binding agent that released the zinc slowly, so it wasn’t irritating to the stomach, and also achieved around the clock plasma zinc elevation.

The study was a placebo controlled 6 month trial in mild to moderate AD patients (Brewer, 2012). Thirty AD patients received 150 mg once daily of the new zinc formulation, and 30 age-matched controls received matching placebo. When giving zinc in high dose like this, one has to be concerned about inducing copper deficiency, which if allowed to become severe, can lead to a severe neurologic syndrome. For that reason we regularly monitored serum Cp, a good measure of copper status, as well as hemoglobin levels, anemia being one of the first manifestations of copper deficiency. During the 6 month study only one patient had zinc dose reduced because of a decreasing Cp. Endpoints were increased serum zinc, decreased serum free copper, and better cognitive scores compared to controls. Cognition was measured by Alzheimer’s Disease Assessment Scale for Cognition (ADAS-cog), Clinical Dementia Rating Scale, Sum of Boxes (CDR-SOB), and Mini Mental State Examination (MMSE).

We anticipated a possible decrease in serum free copper with zinc therapy because zinc blocks the absorption of copper in the intestine. This is the basis of using zinc as a therapy for Wilson’s disease, an inherited disease of copper accumulation and copper toxicity.

We found serum zinc significantly increased and serum free copper significantly decreased in zinc treated patients versus controls. All three cognition scores were in a favorable direction in the zinc treated group versus controls, but none were significant, although CDR-SOB was close at *p* = 0.1. Looking at the data *post hoc*, we realized cognition was relatively stable in controls until age 70, when it began deteriorating much more rapidly, while zinc treated patients remained relatively stable at age 70 and over. We reanalyzed the data on those age 70 and over, and the 15 age matched controls showed significant cognition loss compared to the 14 AD patients, with ADAS-Cog at *p* = 0.037, CDR-SOB at *p* = 0.032, and MMSE close to significant at *p* = 0.067 (Brewer, 2012).

Thus, we conclude from this trial that zinc supplementation will significantly stabilize cognition in AD patients age 70 and over. We believe it will also stabilize cognition in younger patients, but it is harder to show it statistically because controls are deteriorating so slowly before age 70. This study should be confirmed by a larger study, and carried out longer, to evaluate whether the stabilization by zinc therapy is maintained long term.

Assuming zinc therapy has efficacy in this study, the mechanism could be restoration, or partial restoration, of protective zinc in the neuron, or lowering of toxic tree copper levels. Or perhaps both mechanisms are operative since we achieved both. Thus, at this point we can’t be certain that zinc deficiency is a risk factor in AD, because zinc supplementation may be acting through lowering serum free copper.

## Summary

We point out that we have a serious new epidemic of AD, particularly in developed countries, that is robbing a large portion of our elderly of their golden years. We hypothesize that ingestion of inorganic copper from drinking water and supplement pills, together with a high fat diet, are major causal factors in the epidemic. We show that copper from copper plumbing is leached into drinking water at high enough levels to be causal for AD, according to a rabbit AD model. We advise those who believe the hypothesis to be correct to curtail their ingestion of inorganic copper.

We also hypothesize that zinc deficiency is a risk factor for AD, and show that in patients age 70 and over, 6 months of zinc therapy significantly decreased cognition loss compared to age matched controls.

## Conflict of interest statement

The author declares that the research was conducted in the absence of any commercial or financial relationships that could be construed as a potential conflict of interest.
